# Evaluation of Black Soldier Fly Larvae Meal on Growth, Body Composition, Immune Responses, and Antioxidant Capacity of Redclaw Crayfish (*Cherax quadricarinatus*) Juveniles

**DOI:** 10.3390/ani14030404

**Published:** 2024-01-26

**Authors:** Jen-Hong Chu, Tzu-Wei Huang

**Affiliations:** Department of Aquatic Biosciences, National Chiayi University, Chiayi City 600, Taiwan; zoffy80412@gmail.com

**Keywords:** insect meal, Decapoda, fish meal, organic waste

## Abstract

**Simple Summary:**

Due to the increasing price and the availability of fish meal in the future, fish meal is becoming an uneconomic ingredient used in aquafeed. Here, we aimed to use black soldier fly (*Hermetia illucens*) meal as a sustainable protein source in the aquafeed industry. Results showed fully substituting fish meal with black soldier fly (*H. illucens*) meal derived from larvae fed with fishery byproducts considerably improved the growth performance, immune responses, and increased antioxidant enzyme activity of the redclaw crayfish. The enhance black soldier fly nutrition composition as a protein source developed in this study has potential for use in aquafeed in the aquaculture sector.

**Abstract:**

This study investigated the effects of substituting fish meal (FM) with black soldier fly (*Hermetia illucens*) meal (BSM) on the growth performance, body composition, immune response, and antioxidant enzyme activity of juvenile redclaw crayfish (*Cherax quadricarinatus*). Four isonitrogenous (41%) and isolipidic (11%) diets (i.e., FM substitutes) were formulated from BSM prepared using larvae that were fed soybean meal (BSM-S), fishery byproducts (BSM-F), or pitaya (BSM-P). The experimental diets were fed twice daily to triplicate groups of juvenile redclaw crayfish (0.56 ± 0.04 g). After the feed trial, the FM and BSM-F groups exhibited significantly lower feed conversion ratios and significantly higher weight gain; specific growth rates; and concentrations of saturated fatty acids, highly unsaturated fatty acids, eicosapentaenoic acid, and docosahexaenoic acid in the muscle. Among the tested groups, the BSM-F group exhibited significantly enhanced immune responses and increased antioxidant enzyme activity (i.e., superoxide dismutase, phenoloxidase, and glutathione peroxidase); the BSM-P group exhibited a significantly higher feed intake and hepatopancreatic index; and the FM group exhibited a significantly higher muscle body index and apparent digestibility for the dry matter of crude protein. The findings indicate that the juvenile redclaw crayfish fed BSM-F achieved the highest weight gain among the groups.

## 1. Introduction

Protein, the most expensive component of formulated feed, is the primary factor affecting the growth performance of organisms. Because of its high amino acid content, excellent nutrient digestibility, and low antinutritional factor, fish meal (FM) is typically used as the main source of protein in carnivorous fish feed [1]. The current global aquaculture production exceeds the output of natural fisheries, and it is projected to continue growing, which is likely to lead to a gradual increase in the price of FM. Additionally, the use of FM in aquafeed production is associated with various challenges, including an unstable supply and a continually increasing price [2]. Aquafeed accounts for approximately 4.2% of the global compound feed tonnage, which reached 1.266 billion metric tons in 2022 [3]. However, the costs of soybean meal and FM have continually increased [4,5]. Furthermore, their future use in animal feed is expected to be limited because the consumption of soybean and fish products by humans is likely to increase [5]. Therefore, alternative protein sources must be identified to promote sustainable aquaculture development.

As the largest group of faunal species, insects are among the most abundant animals in the world. In Southeast Asian countries, insects are a staple food source because of their high protein content. Specific insects can be bred commercially because of their rapid growth rates, short life cycles, ability to consume various foods, and low costs [6,7,8]. As highlighted previously by [9], nutritionists have proposed including yellow insect meals in aquafeed to increase sustainability without reducing the performance and quality of aquatic organisms. As a food source, the black soldier fly, *Hermetia illucens*, is rich in amino acids (e.g., isoleucine, leucine, lysine, and valine) and fatty acids (e.g., dodecanoic acid, palmitic acid, and tetradecanoic acid) [10]. A few amino acids, such as cystine, methionine, and histidine, are first-limiting amino acids in the black soldier fly meal (BSM) [11,12,13]. The amino acid content in BSM can be adjusted by incorporating various sources of organic waste into the diet of black soldier fly larvae during the rearing period. The use of the black soldier fly as food can potentially reduce food and animal waste and enable the production of animal-grade feedstuff that is high in protein and fat. The nutrition content of the black soldier fly is largely dependent on its diet [14]. A previous study [15] reported that black soldier fly prepupae contain α-linolenic acid, eicosapentaenoic acid (EPA), and docosahexaenoic acid (DHA) when fish offal is included in their diet.

Numerous studies have investigated the use of various insects in aquafeed for different fish species, such as fish and shrimp [11,15,16,17,18,19,20]. Their results indicated that the partial replacement of FM with BSM enhanced the growth parameters of these aquatic organisms. BSM is not only a favorable source of protein but also an immunostimulatory substance because of its chitin content.

Organic waste is produced across all food industries [21]. Most forms of food waste contain a high level of fiber with low digestibility and are poor in nutrients such as protein and vitamins [22]. The byproducts of food industries are typically regarded as refuse; however, various types of food waste contain specific amino acids (e.g., isoleucine, leucine, and methionine) and fatty acids (e.g., linoleic acid, oleic acid, and docosahexaenoic acid) that can be used to produce animal feed [22]. Therefore, numerous studies have investigated the feasibility of using food waste as a food source for different aquatic animals, such as the olive flounder Nile tilapia and Asian sea bass [22,23,24].

The redclaw crayfish (*Cherax quadricarinatus*), also known as the freshwater blueclaw crayfish, is an omnivorous freshwater decapod native to Australia. In 2017, its production value was AUD 423,400 [25]. The redclaw crayfish is regarded as a pivotal mariculture fish species in Taiwan because of its rapid growth and high disease resistance. A previous report [26] estimated that the annual production of redclaw crayfish in Taiwan was approximately 1800 tons. At present, this species is typically fed a commercial diet that was originally designed for Pacific white shrimp (*L. vannamei*) and contains 37% protein and 10% lipid. The composition of this diet is similar to that of the commercial crayfish pellets (>25% protein and >8% lipid) used in several countries, and it has been demonstrated to be effective for improving growth in redclaw crayfish [27]. Because of its high nutritional value and palatability, high-quality FM is used to meet the protein requirements of carnivorous and omnivorous aquatic species [28]. However, the increasing prices and volatile supply of FM have made it a less economical ingredient for aquafeed relative to other options [29,30,31]. Thus, suitable alternatives to FM [32], such as insect protein, are urgently required.

Few studies have investigated the effects of dietary substitutes for FM on the growth performance of redclaw crayfish. The use of insect meal as aquafeed remains an emerging concept, and limited research has been conducted on the topic. Therefore, in the present study, we investigated the effects of substituting FM with BSM on the growth performance, body composition, and digestibility of juvenile redclaw crayfish (*C. quadricarinatus*). Numerous forms of waste are rich in various nutrients; however, because these nutrients cannot be added directly to aquafeed, such waste is discarded. Black soldier larvae were reared to serve as the protein source in the present study.

## 2. Materials and Methods

### 2.1. Experimental Diets

Approximately 210,000 black soldier fly eggs were obtained from Kunyi Biotech (Chiayi County, Taiwan) and were reared at National Chiayi University (NCYU; Chiayi City, Taiwan). The eggs were hatched and grown in empty containers (140 × 100 × 50 cm^3^) indoors, where the temperature was maintained at 22–25 °C. Three days after hatching, the larvae were equally divided into three containers (140 × 100 × 50 cm; number of larvae per container, 70,000) and weighed (average total weight of larvae per container: 4.5 g). The larvae in the containers were randomly assigned to be fed one of the following three experimental diets: soybean meal (crude protein, 38.4%; crude lipid, 17.26%; dry matter; TTET Union Corporation, Tainan City, Taiwan), fishery byproducts (crude protein, 32.7%; crude lipid, 16%; wet weight; Fish Market Processing Industry, Chiayi City, Taiwan), and red-flesh pitaya (crude protein, 1.1%; crude lipid, 0.9%; wet weight; Fruit Market Processing Industry, Pingtung, Taiwan). Before formulation was performed, black soldier larvae in the pupal stage were dried, homogenized, and analyzed for crude protein and crude lipid compositions. The crude protein and crude lipid levels were 50% and 6.9%, respectively, in the black soldier larvae fed soybean meal; 45% and 4.5%, respectively, in the black soldier larvae fed red-flesh pitaya meal; and 55% and 8%, respectively, in the black soldier larvae fed fishery byproduct meal. The chitin content of the BSM used in the present study ranged from 10.31% to 10.75%.

Table 1 lists the ingredients of the experimental diets and the results of proximate analyses. Three isonitrogenous (41%) and isolipidic (11%) diets were formulated by completely substituting FM with BSM-S, BSM-F, or BSM-P. In the experiments, FM, BSM, and casein were the primary sources of protein; fish oil and soybean oil (2:1 [*v*/*v*]) were the sources of lipids; and α-starch served as the carbohydrate source and binder. All dry ingredients were ground into small particles by using a hammer mill, and manual mixing was performed to obtain a homogeneous powder. Subsequently, oil was added to the dry mixture, after which 200 mL of water (per kilogram of the aforementioned powder) was added to form a moist dough. The dough was then cold-extruded through a chopper (diameter, 3 mm) to generate pellets, which were then dried in an oven at 60 °C for 12 h. After cooling was performed, all experimental diets were frozen and stored until use. Before they were used as feed, the prepared foods were crushed and sieved on the basis of the gape of the fish used in the experiments. The gross energy levels of the diets were determined using a bomb calorimeter (IKA calorimeter system, C2000 basic, Staufen, Germany).

### 2.2. Compositions of Fatty Acids and Amino Acids

Table 2 presents the fatty acid and amino acid compositions of the experimental diets. In BSM-S, the predominant fatty acids were linoleic acid (18:2 n-6; 38.27%) and palmitic acid (16:0; 14.48%), and the predominant amino acids were methionine, arginine, and leucine. The total content levels of highly unsaturated fatty acids (HUFAs) and lysine were lower in the BSM-S than in the BSM-F. In the BSM-P, the predominant fatty acids were palmitic acid (16:0; 34.28%) and linoleic acid (18:2 n-6; 26.52%). The content of arginine was higher in the BSM-P than in the BSM-F and BSM-S. In the BSM-F, the predominant fatty acids were linoleic acid (18:2 n-6; 30.24%) and stearic acid (18:0; 20.71%). The content levels of eicosapentaenoic acid (EPA; 20:5 n-3), docosahexaenoic acid (DHA 22:6 n-3), and lysine were higher in the BSM-F than in the BSM-P and BSM-S.

### 2.3. Experimental System

The experiments were conducted at the Aquaculture Research Center of NCYU. The experimental system comprised 12 independent rectangular fiberglass tanks (300 L; 1 × 1 × 0.37 m^3^). Each tank was equipped with an individual pump and an individual water inlet. Water discharged from the fiberglass tanks was sterilized by passing it consecutively through a mechanical filter, a biological filter, and multiple sedimentation tanks under ultraviolet irradiation. Subsequently, the purified water was pumped back into the experimental tanks. The water temperature of the tanks was maintained at 28–30 °C throughout the experimental period. Furthermore, the tanks were maintained under a 12 h light/dark cycle. Each fiberglass tank was supplied with dissolved oxygen through an air stone connected to a central air compressor. Throughout the experimental period, the concentration of dissolved oxygen was maintained at 5.7–6.0 ppm. Furthermore, the pH and salinity, which were measured daily, were maintained at between 7 and 8 and between 33‰ and 35‰, respectively.

### 2.4. Experimental Animal and Feeding Experiment

All animal experiments were conducted in accordance with the guidelines for the use and care of laboratory animals of the Institutional Animal Care and Use Committee of National Chiayi University (NCYU) (approval no. NCYU-110031).

In total, 240 juvenile redclaw crayfish (average weight, 0.56 ± 0.04 g) were purchased from a fish breeding farm in southern Taiwan and were transferred to our laboratory in a 2000 L fiberglass-reinforced plastic barrel. The redclaw crayfish were acclimatized for 2 weeks and fed commercial feed daily (No. 3; Shye Yih Feeding, Kaohsiung City, Taiwan). After starving the redclaw crayfish for 12 h, we randomly distributed them into 12 fiberglass tanks (20 redclaw crayfish per tank). The redclaw crayfish were fed twice daily (8:00 and 15:00) with one of the four experimental diets (FM, BSM-S, BSM-F, or BSM-P) until satiation for 56 days. Three experimental replicates were used. Fecal matter was collected, dried, and stored in a freezer before each feeding session. The amount of feed consumed by the redclaw crayfish in each tank was recorded every week.

### 2.5. Sample Collection

#### 2.5.1. Sample Collection and Analytical Method

At the end of the experiment, the redclaw crayfish were fasted for 24 h. Samples were collected from each replicate of the experimental group. Ten redclaw crayfish were selected through random sampling and frozen until they reached a comatose state. Subsequently, their hepatopancreatic and muscle tissues were dissected to measure the tissues’ hepatopancreatic index (HSI) and muscle body index. Another 10 redclaw crayfish were used to conduct an air exposure challenge. The tissues were oven-dried at 60 °C, homogenized, and stored at −20 °C until further use. In accordance with the standard method established by the Association of Official Agricultural Chemists, the contents of moisture, ash, and crude proteins in the experimental diets were analyzed to determine the approximate composition of each type of feed, and the redclaw crayfish muscle tissues were analyzed using the standard method. Crude protein content was measured using a Kjeltec semiautoanalyzer (model 1007; Foss Tecator, Höganäs, Sweden). The crude lipid content was determined using the chloroform/methanol (2:1 [*v*/*v*]) extraction method as described by a previous report [34]. The crude fiber content was determined through acid and alkali digestion, which was performed using the Fibertec System M1020 (Foss Tecator). Ash and moisture contents were determined using a muffle furnace and an oven (conventional method), respectively.

#### 2.5.2. Growth Parameters

Weight gain (WG) percentage and specific growth rate (SGR) were calculated as follows:

WG% = 100 × (Wt − W0)/W0,

SGR (% day − 1) = 100 × ([ln Wt − ln W0]/t),

where W0 is the initial mean body weight (g), Wt is the final mean body weight (g), and t (days) is the growing period.

The feed conversion ratio (FCR) and survival rate were calculated as follows:

FCR = feed intake (FI [g])/WG (g).

The protein efficiency ratio (PER) was calculated by determining the relationship between the increase in the body weight of redclaw crayfish and the amount of protein that they consumed.

PER = weight gain of redclaw crayfish/crude protein in diet

Survival rate (%) = 100 × (final number of redclaw crayfish/initial number of redclaw crayfish)

After the redclaw crayfish were euthanized through immersion in ice-cold water, hepatopancreatic tissues were harvested from 10 from each experimental group to determine their HSI levels. The redclaw crayfish muscle tissues were carefully dissected, dried, homogenized, and frozen for subsequent proximate analyses.

#### 2.5.3. Analyses of Fatty Acids and Amino Acids

Fatty acids were analyzed using the method described by a previous report [35]. In brief, saponified lipids were methylated per the method described by another previous report [36]; that is, refluxing was performed for 20 min in 2 mL of 14% boron trifluoride in methanol. The resultant fatty acid methyl esters were analyzed through gas–liquid chromatography, which was performed using a Trace GC 2000 instrument (Spectra Lab Scientific, Markham, ON, Canada) equipped with a flame ionization detector. Subsequently, these esters were separated using a Restek capillary column (30 m × 0.28 mm; film thickness, 0.25 μm; Stabilwax; isotherm, 208 °C). The temperatures of the injector and detector were maintained at 250 °C and 200 °C, respectively. Nitrogen was used as a carrier gas. Fatty acids were identified by comparing their retention times with those of a reference sample (GLC-68A; Nu-Chek-Prep, Elysian, MN, USA) comprising a mixture of saturated and unsaturated fatty acids. Chromatogram peaks were compared with the peaks obtained for a sample of cod liver oil, which served as a secondary reference sample.

The amino acid compositions of the experimental diets were determined using the standard method established by the Association of Official Agricultural Chemists (994.12 Amino Acid in Feeds). Each sample was freeze-dried and degreased, after which 1 mL of 6-N HCl solution containing 1% phenol was added. Next, nitrogen was pumped to remove air from the samples and to seal them. Subsequently, to hydrolyze the acids, the mixture was heated at 105 °C for 24 h and filtered using a 0.22 μm syringe filter. o-phthalaldehyde was added at a ratio of 1:1 to the filtrate. Amino acids were analyzed through high-performance liquid chromatography (Hitachi L-8900). The samples were separated on a Gemini 5u NX-C18 column (150 × 4.6 mm^2^; 110 A; 40 °C) equipped with an incubator (Super Co-150; Enshine, Taipei, Taiwan). A mixture of phosphoric acid, tetrahydrofuran, and methanol (ratio, 960:20:20; 50 mM) was used as mobile phase A, and a mixture of methanol and water (ratio, 65:35) was used as mobile phase B. The flow rate was set at 1 mL/min, and the excitation and emission wavelengths were set at 340 and 455 mm, respectively.

#### 2.5.4. Digestibility Analysis

To analyze digestibility, the diets were supplemented with chromic oxide as an inert marker. Fecal samples were collected from the fiberglass tanks through filtration (Whatman No. 2); they were freeze-dried and placed in plastic bags at −40 °C for analysis. Next, 65% HNO_3_ (Fulka, Muskegon, MI, USA) was mixed with 3 mL of 30% H_2_O_2_ (Merck, Darmstadt, Germany) and used for sample digestion in a microwave (Multiwave Go; Anton Paar, Graz, Austria). The total chromic oxide concentration in the samples was determined through inductively coupled plasma optical emission spectroscopy.

The apparent digestibility of dry matter of protein (ADp) was calculated as follows:

ADp = (1 − [*C*_d_/*C*_f_] × [NX_f_/NX_d_]) × 100,

where d is the diet, f is the feces, *C* is the chromic oxide concentration, and NX is the nutrient concentration.

#### 2.5.5. Nitrogen Retention Efficiency

Nitrogen retention efficiency (RN) = ([N56 − N0]/56 × 1000)/Nd × 100,

where N56 and N0 are the nitrogen content in the redclaw crayfish at harvest and stocking (g), respectively. N56 = W56 × N redclaw crayfish (56); N0 = W0 × N redclaw crayfish (0). Nd represents the amount of nitrogen digested.

#### 2.5.6. Air Exposure Challenge

The air exposure challenge was performed in accordance with the modified procedures described by a previous report [37]. The 8 h air exposure challenge trial was conducted at three temperatures (30 °C, 35 °C, and 40 °C), and it revealed the following results. The survival rates of the redclaw crayfish treated at 30 °C and 35 °C were 96% and 98%, respectively; by contrast, this rate was significantly higher (54%) when the treatment was conducted at 40 °C (*p* < 0.05). On the first day after the feeding trial, 10 redclaw crayfish from each tank were exposed to warm air (35 °C) for 8 h. No mortality was observed among the redclaw crayfish throughout the 8 h air exposure challenge. After conducting the challenge, we analyzed the hepatopancreatic tissue samples for phenoloxidase (PO), superoxide dismutase (SOD), glutathione peroxidase (GP_X_), respiratory burst, and lysozyme activity. These results were used to evaluate the biological and immune responses of the redclaw crayfish, which were fed various experimental diets, to the air exposure challenge.

In the present study, PO activity was spectrophotometrically measured in accordance with the modified Coomassie blue staining procedure as described by a previous report [38]. Coelomic fluid samples from the experimental redclaw crayfish were homogenized in a homogenizer (PREMA, IADI-HG-300D). These samples were placed in a 5 mL Eppendorf tube and centrifuged at 2500× *g* for 15 min at 4 °C. The resulting supernatant was collected for further analysis. To measure PO activity, 100 μL of a coelomic fluid sample was placed in 96-wellmicrotiter plates and incubated for 30 min with 50 μL of L-3,4 dihydroxy phenyl alanine (3 mg/mL). Optical density was measured at the wavelength of 492 nm using a microplate reader. In the present study, enzyme activity is expressed as the change in absorbance per minute per 100 μL of coelomic fluid.

An SOD assay was conducted using a Ransod kit (Randox Laboratories, Crumlin, UK) in accordance with the manufacturer’s instructions. Hepatopancreatic samples weighing 0.2 g were homogenized for 5 min with 2.7 mL of phosphate buffer containing 1% Triton X-100 (0.05 M, pH 7.8). The resulting cell suspension was transferred to tubes and centrifuged at 13,000× *g* for 30 min at 4 °C. Subsequently, 25 μL of muscle tissue solution or Hank’s balanced salt solution (as a control) was added to 850 μL of a reaction substrate containing xanthine and 2-(4-iodophenyl)-3-(4-nutrophenol 3-5-phenltetrazolium, after which 125 μL of xanthine oxidase was added. The decrease in absorbance at 505 nm was recorded for the durations of 30 and 210 s. Specific activity was defined as the amount of SOD resulting in a 50% reduction in the rate of formazan dye formation.

A glutathione peroxidase (GP_X_) assay was conducted using a colorimetric assay kit (ab102530) in accordance with the manufacturer’s instructions. After hepatopancreatic tissue samples were extracted, 0.2 g of hepatopancreatic tissue was placed in liquid nitrogen and immediately stored at −80 °C. Each 100 mg aliquot of tissue sample was rinsed with cold PBS, resuspended in 200 µL of cold assay buffer, and transferred to tubes where it was centrifuged at 10,000× *g* for 15 min at 4 °C. A 50 μL sample of hepatopancreatic tissue solution or a 100 µL standard dilution solution (as a control) was added to 40 µL of the resulting reaction mixture; the sample solution and control solution were then placed in positive control or reagent control wells, respectively. The samples were thoroughly mixed and incubated at room temperature for 15 min to deplete all glutathione disulfide. Subsequently, 10 µL of cumene hydroperoxide solution was added. The decrease in absorbance at 340 nm was monitored using a microplate reader, after which the sample was incubated at 25 °C for 5 min. Absorbance was measured using a microplate reader at 340 nm. The samples that produced signals exceeding that of the highest standard were further diluted in an appropriate buffer and reanalyzed. The final concentration was calculated by multiplying the sample concentration by the corresponding dilution factor.

Blood was collected from the sinusoid by using a syringe and centrifuged at 300× *g* for 20 min at 4 °C. The resulting sample was used to analyze immune indicators. In accordance with the method described by a previous report [39], respiratory burst activity was determined by examining the reduction of nitroblue tetrazolium (NBT) to formazan as a measure of intracellular superoxide production. In brief, 100 μL of hemolymph and 100 μL of poly-L-lysine solution (0.2%) were added to 96-microplate wells (Nunclon Surface, Roskilde, Denmark) and incubated for 30 min to improve cell adhesion. The microplates were then centrifuged at 300× *g* for 15 min. After centrifugation, plasma was removed from the microplates, and 100 μL of zymogen solution was added. The resulting mixture was allowed to react for 30 min at room temperature. The resulting hemocyte samples were washed three times with modified complete Hank’s balanced salt solution and stained with 100 μL of NBT solution (0.3% in distilled water) for 30 min at room temperature. The reaction was stopped by adding 100 μL of methanol (100%), after which the hemocyte samples were washed three times with 100 μL of methanol (70%). After air drying was performed, formazan was dissolved by adding 120 μL of KOH and 150 μL of dimethyl sulfoxide for 2 min. In the present study, respiratory burst activity is expressed as the NBT reduction measured at an optical density of 360 nm.

Lysozyme activity was measured in accordance with the method as described by a previous report [40]. In brief, 10 μL of serum from a redclaw crayfish was added to a 96-well microplate, after which 200 μL of *Micrococcus lysodeikticus* was added. After the resulting mixture underwent rapid mixing, an ELISA plate reader was used to measure the turbidity of the mixture every 1 min for 6 min at 530 nm. One unit of lysozyme activity was defined as the amount of enzyme resulting in a 0.001 decrease in absorbance per minute per milliliter of serum. The level of lysozyme activity was calculated using a standard curve derived from chicken egg white lysozyme (L4631-IVL, Sigma-Aldrich, Darmstadt, Germany).

### 2.6. Statistical Analysis

All experimental data underwent one-way analysis of variance at a significance level of 0.05. Statistical analyses were performed using SAS for Windows (SAS Institute, Cary, NC, USA). Whenever a significant difference (*p* < 0.05) was identified, Tukey’s range test was performed to identify significant differences among the experimental groups [41].

## 3. Results

### 3.1. Growth Performance and Muscle Composition

Table 3 presents the results regarding the growth performance of the juvenile redclaw crayfish that were fed different experimental diets for 56 days. The experimental redclaw crayfish had a survival rate of 100%. The final weight, WG, SGR, and PER of the FM and BSM-F groups were significantly higher than those of the BSM-S and BSM-P groups (*p* < 0.05). The BSM-F and BSM-P groups exhibited the lowest and highest FCRs, respectively.

Table 4 presents the results of a proximate analysis of redclaw crayfish muscle tissues. The BSM-P group exhibited lower crude protein content than did the other groups. The crude protein content in the muscle was highest in the FM group, followed by that in the BSM-F group. The crude lipid content in the muscle ranged from 1.83% to 1.85% among the groups. The contents of moisture and ash in the muscle were the highest in the BSM-P group (moisture, 78.13%; ash, 0.37%).

### 3.2. Feed Utilization

Table 5 presents the FI, HSI, muscle body index (MBI), ADp, and RN values of the experimental groups. The FI was significantly higher in the BSM-P group (108.87 g/tank) than in the other groups (*p* < 0.05). The MBI was significantly higher in the FM group (81.38 ± 2.20%) than in the experimental groups (*p* < 0.05). Furthermore, the MBI was significantly higher in the BSM-F group than in the BSM-S and BSM-P groups (*p* < 0.05). The HSI was significantly higher in the BSM-P group than in the other groups (*p* < 0.05) but significantly lower in the FM group than in the other experimental groups (*p* < 0.05).

The ADp was significantly lower in the BSM-S and BSM-P groups than in the FM and BSM-F groups (*p* < 0.05). The RN was not significantly different among the four groups (*p* > 0.05).

Table 6 presents the muscle amino acid compositions of the redclaw crayfish. The muscle lysine level of the redclaw crayfish fed SBM-F was significantly lower than that of the redclaw crayfish fed FM but significantly higher than those of the redclaw crayfish fed SBM-S or SBM-P (*p* < 0.05). The methionine, threonine, and tryptophan levels of the muscle of the redclaw crayfish fed FM were not significantly different from those of the redclaw crayfish fed SBM-F but were significantly higher than those of the redclaw crayfish fed SBM-S or SBM-P (*p* < 0.05). The total essential amino acids (EAAs) in the redclaw crayfish muscle contributed 6.65 to 8.04 g kg^−1^ to the total protein.

Table 7 presents the muscle fatty acid compositions of the redclaw crayfish. The contents of EPA, docosapentaenoic acid (22:5 n-3), and DHA in the muscle were significantly higher in the FM group than in the experimental groups (*p* < 0.05). The contents of EPA and DHA were significantly higher in the BSM-F group than in the BSM-S and BSM-P groups. The contents of oleic, linoleic, and α-linolenic acids were significantly higher in the BSM-S group than in the other experimental groups.

### 3.3. Immune Response and Antioxidant Enzyme Activity

Table 8 lists the SOD, PO, and GP_X_ activities of the hepatopancreatic tissue of the juvenile redclaw crayfish that were fed the experimental diets for 56 days. The SOD (0.29 U min^−1^) and PO (0.36 U min^−1^) activities of the juvenile redclaw crayfish that were fed BSM-F were significantly higher than those of the juvenile redclaw crayfish that were fed the other diets (*p* < 0.05). The GP_X_ activity (0.32 mU mL^−1^) of the juvenile redclaw crayfish that were fed the BSM-F was significantly higher than that of the redclaw crayfish that were fed BSM-S or BSM-P (*p* < 0.05) but not significantly higher than that of the redclaw crayfish that were fed FM. The analysis of variance results indicated that the interaction between exposure challenge and treatment had a significant effect on the SOD activity of the redclaw crayfish larvae.

**Figure 1:**
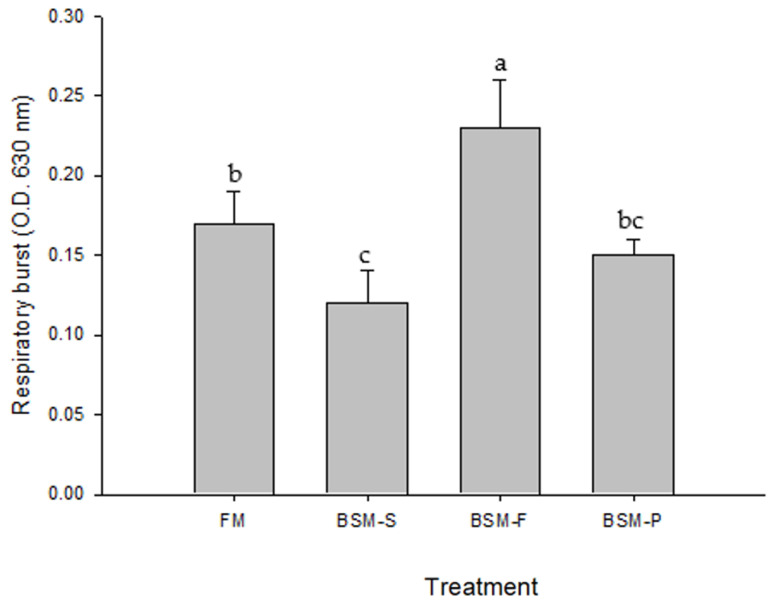
Hemocyte respiratory burst of redclaw crayfish fed different experimental diets for 56 days. Data are expressed as mean ± standard error values (10 redclaw crayfish per tank; *n* = 3), and different letters indicate significant differences among groups (*p* < 0.05).

**Figure 2:**
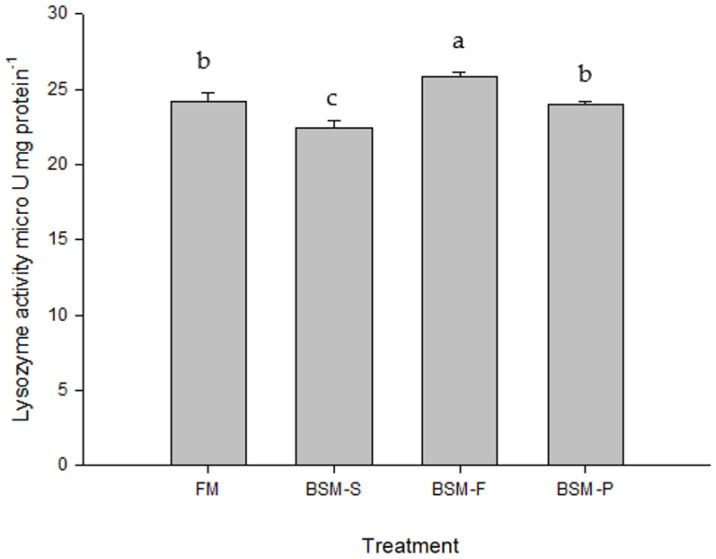
Hemocyte lysozyme activity of redclaw crayfish fed different experimental diets for 56 days. Data are expressed as mean ± standard error values (10 redclaw crayfish per tank; *n* = 3), and different letters indicate significant differences among groups (*p* < 0.05).

Figure 1 and Figure 2 list the hemocyte respiratory burst and lysozyme activity of redclaw crayfish fed different experimental diets for 56 days, respectively. We identified enhanced lysozyme activity after the juvenile redclaw crayfish were fed BSM-F. On the basis of their ability to enhance innate immunity, BSM-F was used as a feed protein source in the experiments. The BSM-F group exhibited significantly enhanced respiratory burst activity (0.23) relative to that in the other groups (*p* < 0.05; Figure 1). Figure 2 presents the results pertaining to the lysozyme activities of the hemocytes derived from the redclaw crayfish. The lysozyme activity (25.8 μU/mg protein^−1^) of the juvenile redclaw crayfish that were fed BSM-F was significantly higher than that of the juvenile redclaw crayfish that were fed the other diets (*p* < 0.05).

## 4. Discussion

In the present study, the FI of the redclaw crayfish fed the BSM diets was significantly different from that of the redclaw crayfish fed FM. This finding highlights problems related to the essential nutritional components of the BSM diets and indicates that they were not accepted by the redclaw crayfish. The contents of various amino acids (e.g., lysine, leucine, and valine) and fatty acids (e.g., EPA and DHA) were substantially lower in the black soldier fly larvae fed plant-based ingredients than in those fed FM. This finding indicates that the juvenile redclaw crayfish that were fed BSM-S or BSM-P exhibited the lowest growth and feed utilization. Because of their antinutritional value, lack of specific EAAs, low bioavailability, and low palatability, plant-based ingredients provide fewer benefits than FM does [42]. The higher dietary total EAA content of FM relative to that of the flies fed plant-based ingredients contributed to the higher growth and free EAA content of muscle in the redclaw crayfish that were fed FM. This higher growth can be explained by the balance between dietary essential amino acid profiles and efficiency of protein utilization affecting overall animal performance [43]. Another possible explanation for the higher growth is the differences in the fatty acid compositions of BSM-F and FM; DHA has been reported as the first-limiting fatty acid in numerous aquatic organism diets [15]. The essential fatty acid contents of soybean meal and pitaya were inadequate to meet the nutritional requirements of the redclaw crayfish; this may explain the lower average weight of the redclaw crayfish fed BSM-S or BSM-P compared with the weights of those fed FM or BSM-F. A previous study [44] reported that juvenile redclaw crayfish fed the diet supplemented with vegetable oil had lower DHA and EPA contents than those fed diet supplemented with fish oil. Furthermore, substituting FM with BSM-F instead of BSM-S or BSM-P as the sole protein source resulted in more favorable growth and feed performance in the redclaw crayfish. Several studies have reported similar findings with respect to the use of BSM-F as feed for fish and shrimp species [12,16,18,39,45,46]. The conclusion of the feeding trial in the present study was that no mortality was observed among the examined redclaw crayfish, irrespective of their diet (control or treatment).

In terms of the growth and feed utilization efficiency of the redclaw crayfish, more favorable results were achieved by feeding them FM or BSM-F than by feeding them BSM-S or BSM-P. The DHA, EPA, and lysine levels were lower in the BSM-S and BSM-P groups than in the FM and BSM-F groups, indicating that the dietary protein derived from BSM-S and BSM-P was insufficient to replace that provided by FM in the diet of juvenile redclaw crayfish. Similar improvements in nutrition composition have also been reported for black soldier larvae that were fed fish offal [15] and black soldier larvae cultured to the prepupal stage [12]. In the present study, the redclaw crayfish fed BSM-F exhibited higher HUFA levels relative to those of the redclaw crayfish fed BSM-S or BSM-P. The breeding method may have enhanced the nutrient utilization of BSM. Lower essential fatty acid content can inhibit the growth parameters of aquatic organisms [47,48], thereby significantly increasing the nutrition available for digestion and, possibly, improving the nutrient absorption of a diet [15]. Black soldier larvae are viable resources for reducing animal waste and recycling nutrients (e.g., proteins and lipids) and for enhancing the nutritional value of animal diets. Therefore, they are a suitable substitute for FM and fish oil [15]. Numerous studies have indicated that the inclusion of black soldier larvae in the diets of various commercially bred species (e.g., poultry, catfish, tilapia, and swine) effectively increased their omega-3 fatty acid content and enhanced their growth parameters [16,49]. In the present study, the crude lipid content in the muscle tissues of the redclaw crayfish fed BSM was higher than that in the muscle tissues of the redclaw crayfish fed FM. A previous study [50] reported that increasing the quantity of mealworms in the diet of Pacific white shrimp considerably increased their whole-body crude lipid content. Studies on African catfish and Pacific white shrimp have revealed that using mealworm meal as a dietary supplement led to a substantial increase in the lipid content of fish carcasses [51,52]. This finding can be attributed to the imbalanced fatty acid composition of mealworm feed.

The crude protein content in the muscle was significantly higher in the FM and BSM-F groups than in the BSM-P group. These differences in body composition may be attributable to the differences in diet composition. The nutritional composition of the tissues in aquatic organisms is affected by various nutrients, such as proteins, lipids, and carbohydrates [53,54,55]. In the present study, the crude protein content in the BSM-P group was significantly lower than those in the other experimental groups after the experiment. The BSM-P group exhibited increased crude lipid and moisture contents. Other studies have reported a similar increase in lipid content in aquatic organisms fed diets containing plant-derived oils or glucose [56,57,58]. The HSI, which is the ratio of hepatopancreatic weight to body weight, is commonly used as an indicator of lipid reserves in the hepatopancreas [59,60]. A previous study [59] reported that dietary glucose derived from pitaya promoted the synthesis of fatty acids in pinnate batfish and increased their HSI by affecting their levels of glycogen and glucose-6-phosphate dehydrogenase. A previous study [60] reported that the sugars present in pitaya primarily comprise glucose (60–65 mg/g), fructose (28–40 mg/g), and sucrose (1.8–2.5 mg/g). These findings may explain the higher HSI of the BSM-P group relative to those of the other experimental groups.

The redclaw crayfish that were fed BSM-F for 8 weeks exhibited significantly higher respiratory burst and lysozyme activities than did those that were fed the other diets. This finding indicates that BSM-F has immunostimulatory effects on redclaw crayfish. The immunostimulatory effects of immunostimulants, such as chitosan and chitin, have been examined in decapods [61,62,63]. A previous study [64] reported that black soldier larvae exhibit a higher chitin content in the pupal stage (11%) than in other stages. A previous study [65] indicated that the partial replacement (up to 50%) of dietary FM protein with black soldier fly larvae meal did not negatively affect the health of European seabass; instead, it provided the potential benefits of enhanced antioxidative status and immune responses. Therefore, in the present study, the improved immune enzyme activity of the juvenile redclaw crayfish that were fed BSM may be attributed to the chitin present in black soldier fly pupae. This result aligns with those of studies dealing with decapods [66,67]. However, the growth of the redclaw crayfish was negatively affected when they were fed BSM-S or BSM-P. Several studies have indicated that the immune system of shrimp can be negatively affected when they are fed nutritionally imbalanced diets (e.g., diets with excessive fat levels or unbalanced amino acids) [68,69]. The findings of the present study indicate that the potential of BSM protein as a substitute for fish protein is limited by its nutritional composition.

Organic waste is typically collected alongside other municipal solid waste and is sent to landfills or incinerators for disposal. Because landfilling, incineration, and the release of organic waste into drainage incur high costs and contribute to environmental contamination and pollution [70], organic waste recycling is becoming an increasingly crucial and studied topic. Our study findings indicate that the juvenile redclaw crayfish that were fed BSM-F did not exhibit significantly different growth parameters relative to those of the juvenile redclaw crayfish fed with FM. The cost of feed for the juvenile redclaw crayfish decreased when the amount of BSM-F used in place of FM increased. Compared with the use of FM, the use of organic waste to cultivate black soldier larvae for aquafeed can lead to reduced feed costs, increased profits, and increased sustainable utilization of organic waste; this finding aligns with previous reports [71,72].

## 5. Conclusions

The body composition of black soldier larvae is dependent on their rearing conditions. Fully substituting FM with BSM derived from larvae fed with soybean and pitaya considerably reduced the growth performance of the redclaw crayfish and changed their muscle lipid contents and fatty acid composition. By contrast, BSM derived from larvae reared on a diet of fishery byproducts served as an effective substitute for FM in the diet of redclaw crayfish without adversely affecting their growth performance.

## Figures and Tables

**Table 1:** Compositions of experimental diets.

Ingredients (Percent Dry Basis)	Diets			
	FM	SBM-S	SBM-F	SBM-P
FM	55			
BSM-S		58.2		
BSM-F			55.1	
BSM-P				66
α-starch ^1^	5	5	5	5
Yeast ^2^	1	1	1	1
Oil ^3^	7.68	7.83	7.75	8.23
Chromic oxide	0.5	0.5	0.5	0.5
Casein	15	15	15	15
Vitamin premix ^4^	2	2	2	2
Mineral premix ^5^	1	1	1	1
Cholesterol	0.5	0.5	0.5	0.5
Vitamin A	0.1	0.1	0.1	0.1
Vitamin D	0.1	0.1	0.1	0.1
Vitamin E	0.1	0.1	0.1	0.1
α-cellulose	12.02	8.67	11.85	0.47
Analyzed composition ^6^				
Moisture	9.88	10.14	9.87	9.98
Crude protein	42.25	41.10	42.31	41.70
Crude lipids	12.25	11.85	12.16	11.2
Crude fiber	13.39	13.91	16.81	6.41
Ash	7.87	8.11	8.88	9.01
NFE ^7^	24.24	25.04	19.85	31.68
Dry matter	90.12	89.86	90.13	90.02
Metabolizable energy	336.79	332.16	320.87	352.03
Gross energy (Kcal/100 g)	376.17	371.16	358.03	394.32

^1^ α-starch (Thai Wah, Bangkok, Thailand). ^2^ Yeast (Sigma-Aldrich, St. Louis, MO, USA). ^3^ Fish oil/soybean oil = 2:1 (*v*/*v*). ^4^ 0.5% thiamine HCl, 0.8% riboflavin, 2.6% niacinamide, 0.1% D-biotin, 1.5% Ca-pantothenate, 0.3% pyridoxine HCl, 0.5% folic acid, 18.1% inositol, 12.1% ascorbic acid, 0.1% cyanocobalamin, 3% para-aminobenzoic acid, 0.1% butylated hydroxytoluene, and 60.3% cellulose. ^5^ Modified from the premix used in another study [33]. ^6^ Expressed in terms of the percentage of dry weight. ^7^ Nitrogen-free extract (NFE) = (100 − [crude protein + crude lipids + crude fiber + ash]) (%). Abbreviations: BSM-S, black soldier larvae fed soybean meal; BSM-F, black soldier larvae fed fishery byproducts; BSM-P, black soldier larvae fed pitaya; FM, fish meal.

**Table 2:** Amino acid (g kg^−1^ of total protein) profiles and fatty acid (percentage of total fatty acid) compositions of experimental diets.

Composition	Ingredients			
	Fish Meal	SBM-S	SBM-F	SBM-P
Amino acid				
Arginine	2.39	0.99	1.02	1.68
Histidine	1.24	0.54	0.64	0.52
Isoleucine	2.43	0.60	0.78	0.79
Leucine	3.93	0.95	1.17	1.29
Lysine	3.69	0.66	1.91	1.07
Methionine	1.40	1.04	1.42	1.32
Phenylalanine	2.20	0.61	0.75	1.10
Threonine	1.91	0.50	0.63	0.65
Valine	2.79	0.79	1.08	1.10
Tryptophan	0.45	0.43	0.49	0.21
ΣEAA	22.43	7.11	9.89	9.73
Fatty acid (%)				
12:0	5.3	2.32	2.47	0.98
14:0	9.75	1.23	1.59	1.07
16:0	23.21	14.48	14.95	34.28
16:1	2.12	1.38	1.85	1.17
18:0	6.9	14.46	20.71	7.44
18:1	1.91	8.97	5.55	10.72
18:2 n-6	24.04	38.27	30.24	26.52
18:3 n-3	3.8	4.65	3.54	4.85
20:0	1.85	1.24	1.66	1.07
20:1	1.84	1.28	1.67	1.12
20:5 n-3	2.37	1.47	2.03	1.23
22:0	1.91	1.2	1.66	2
22:6 n-3	14.92	9.0	12.07	7.5
SFAs	48.92	34.93	43.04	46.84
MUFAs	5.87	11.63	9.07	13.01
PUFAs	27.84	42.92	33.78	31.37
HUFAs	17.29	10.47	14.10	8.73

Fatty acid content was measured in terms of the percentage of total fatty acid content. Abbreviations: SFAs, saturated fatty acids; MUFAs, monounsaturated fatty acids; PUFAs, polyunsaturated fatty acids (two to three unsaturated bonds); HUFAs, highly unsaturated fatty acids (four or more unsaturated bonds); ΣEAA, total essential amino acids.

**Table 3:** FBW, WG, SGR, and PER of juvenile redclaw crayfish samples fed different diets.

Diet	FBW ^1^	WG ^2^	SGR ^3^	PER ^4^
FM	3.58 ± 0.10 ^a^	539.56 ± 18.25 ^a^	3.31 ± 0.05 ^a^	2.30 ± 0.12 ^a^
BSM-S	3.03 ± 0.17 ^b^	442.12 ± 11.90 ^b^	3.02 ± 0.04 ^b^	1.59 ± 0.09 ^b^
BSM-F	3.54 ± 0.09 ^a^	532.98 ± 15.51 ^a^	3.29 ± 0.04 ^a^	2.47 ± 0.12 ^a^
BSM-P	2.23 ± 0.32 ^c^	303.21 ± 52.94 ^c^	2.48 ± 0.23 ^c^	0.86 ± 0.29 ^c^

Values in the same column with different superscripts are significantly different (*p* < 0.05). Data are presented as mean ± standard error values (*n* = 3). ^a, b, c^ Mean values in the same column with different letters are significantly different (*p* < 0.05). ^1^ FBW (g^−1^), final mean weight of juvenile redclaw crayfish. ^2^ WG (%) = (FBW − IBW)/IBW × 100. ^3^ SGR (% day^−1^) = (ln[FBW/IBW])/56 × 100. ^4^ PER = weight gain of redclaw crayfish/crude protein in diet. Abbreviations: FBW, final body weight; WG, weight gain; SGR, specific growth rate; PER, protein efficiency ratio; BSM-S, black soldier larvae fed soybean meal; BSM-F, black soldier larvae fed fishery byproducts; BSM-P, black soldier larvae fed pitaya; FM, fish meal.

**Table 4:** Muscle composition (%, wet weight) of juvenile redclaw crayfish fed different diets for 56 days.

Diet	Composition			
	Moisture	Crude Protein	Crude Lipids	Ash
FM	77.12 ± 0.08 ^c^	18.86 ± 0.02 ^a^	1.85 ± 0.01	0.31 ± 0.01 ^b^
BSM-S	78.05 ± 0.06 ^a^	17.92 ± 0.13 ^c^	1.83 ± 0.01	0.31 ± 0.02 ^b^
BSM-F	77.76 ± 0.17 ^b^	18.24 ± 0.05 ^b^	1.85 ± 0.01	0.30 ± 0.03 ^b^
BSM-P	78.13 ± 0.01 ^a^	17.59 ± 0.03 ^d^	1.83 ± 0.01	0.37 ± 0.01 ^a^

Values in the same column with different superscripts are significantly different (*p* < 0.05). Data are presented as mean ± standard error values (*n* = 3). Abbreviations: BSM-S, black soldier larvae fed soybean meal; BSM-F, black soldier larvae fed fishery byproducts; BSM-P, black soldier larvae fed pitaya; FM, fish meal.

**Table 5:** FI (g), HSI (%), MBI (%), AD (%), ADp (%), and RN (%) of juvenile redclaw crayfish fed different diets for 56 days.

Diet	Index				
	FI ^1^	HIS ^2^	MBI ^3^	ADp ^4^	RN ^5^
FM	69.08 ± 1.49 ^bc^	0.52 ± 0.01 ^d^	81.38 ± 2.20 ^a^	84.87 ± 0.74 ^a^	52.62 ± 3.30
BSM-S	82.42 ± 1.12 ^b^	0.74 ± 0.02 ^b^	65.18 ± 1.54 ^c^	78.09 ± 0.75 ^b^	49.48 ± 5.44
BSM-F	64.88 ± 2.45 ^c^	0.66 ± 0.01 ^c^	75.88 ± 0.26 ^b^	83.18 ± 0.97 ^a^	51.46 ± 2.04
BSM-P	108.87 ± 15.03 ^a^	0.77 ± 0.01 ^a^	63.64 ± 3.16 ^c^	75.62 ± 0.99 ^c^	47.02 ± 2.84

^a, b, c, d^ Mean values in the same column with different letters are significantly different (*p* < 0.05). Data are presented as mean ± standard error values (*n* = 3). ^1^ FI = feed intake (g, dry matter). ^2^ HSI = hepatopancreatic weight/body weight × 100. ^3^ MBI = muscle weight/body weight × 100. ^4^ ADp = (1 − [*C*_d_/*C*_f_] × [NX_f_/NX_d_]) × 100, where d is the diet, f is the feces, *C* is the chromic oxide concentration, and NX is the nutrient concentration. ^5^ RN = ([N56 − N0]/56 × 1000)/Nd × 100. Abbreviations: FI, feed intake; HSI, hepatosomatic index; MBI, muscle body index; ADp, apparent digestibility of crude protein; RN, nitrogen retention efficiency; BSM-S, black soldier larvae fed soybean meal; BSM-F, black soldier larvae fed fishery byproducts; BSM-P, black soldier larvae fed pitaya; FM, fish meal.

**Table 6:** Muscle amino acid (g kg^−1^ total protein) profiles.

Composition	Ingredients			
	Fish Meal	SBM-S	SBM-F	SBM-P
Arginine	1.59 ± 0.23 ^a^	1.34 ± 0.02 ^b^	1.33 ± 0.02 ^b^	1.32 ± 0.03 ^b^
Histidine	0.37 ± 0.02	0.37 ± 0.02	0.35 ± 0.01	0.34 ± 0.02
Isoleucine	0.68 ± 0.01 ^a^	0.61 ± 0.02 ^b^	0.64 ± 0.03 ^ab^	0.62 ± 0.03 ^b^
Leucine	1.17 ± 0.01 ^a^	0.95 ± 0.01 ^c^	1.01 ± 0.02 ^b^	0.89 ± 0.01 ^a^
Lysine	1.24 ± 0.01 ^a^	0.88 ± 0.01 ^c^	1.11 ± 0.10 ^b^	0.87 ± 0.02 ^c^
Methionine	0.37 ± 0.02 ^a^	0.37 ± 0.02 ^a^	0.37 ± 0.03 ^a^	0.31 ± 0.01 ^b^
Phenylalanine	0.66 ± 0.03 ^ab^	0.61 ± 0.02 ^ab^	0.77 ± 0.18 ^a^	0.58 ± 0.01 ^b^
Threonine	0.68 ± 0.01 ^a^	0.55 ± 0.04 ^b^	0.69 ± 0.02 ^a^	0.59 ± 0.01 ^b^
Valine	0.57 ± 0.04 ^a^	0.51 ± 0.01 ^b^	0.54 ± 0.03 ^ab^	0.51 ± 0.02 ^b^
Tryptophan	0.71 ± 0.01 ^a^	0.67 ± 0.02 ^b^	0.71 ± 0.02 ^a^	0.62 ± 0.02 ^c^
ΣEAA	8.04 ± 0.32 ^a^	6.86 ± 0.09 ^c^	7.52 ± 0.28 ^b^	6.65 ± 0.10 ^c^

^a, b, c,^ Mean values in the same row with different letters are significantly different (*p* < 0.05). Data are presented as mean ± standard error values (*n* = 3).

**Table 7:** Major fatty acid contents in muscle tissue of juvenile redclaw crayfish fed different diets for 56 days.

Fatty Acid (%)	Treatment			
	FM	BSM-S	BSM-F	BSM-P
14:0	7.05 ± 0.10 ^a^	5.15 ± 0.09 ^c^	6.10 ± 0.09 ^b^	4.67 ± 0.08 ^d^
14:1	3.46 ± 0.26 ^c^	6.26 ± 0.48 ^a^	4.86 ± 0.37 ^b^	6.96 ± 0.54 ^a^
16:0	22.60 ± 0.27 ^c^	24.24 ± 0.51 ^b^	22.92 ± 0.39 ^c^	25.34 ± 0.53 ^a^
16:1	9.55 ± 0.30 ^a^	7.36 ± 0.58 ^c^	8.45 ± 0.44 ^b^	6.81 ± 0.66 ^c^
18:0	4.76 ± 0.12 ^d^	6.50 ± 0.20 ^b^	5.63 ± 0.16 ^c^	6.94 ± 0.22 ^a^
18:1	6.20 ± 0.40 ^b^	7.82 ± 0.73 ^a^	7.01 ± 0.56 ^ab^	8.22 ± 0.81 ^a^
18:2 n-6	1.88 ± 0.02 ^d^	3.40 ± 0.11 ^a^	2.05 ± 0.03 ^c^	2.30 ± 0.04 ^b^
18:3 n-3	0.45 ± 0.02 ^c^	0.53 ± 0.02 ^a^	0.49 ± 0.02 ^b^	0.55 ± 0.02 ^a^
20:0	3.12 ± 0.01 ^a^	1.94 ± 0.01 ^c^	2.53 ± 0.01 ^b^	1.64 ± 0.01 ^d^
20:1	5.50 ± 0.29 ^d^	9.26 ± 0.52 ^b^	7.38 ± 0.40 ^c^	10.19 ± 0.57 ^a^
20:2 n-6	1.71 ± 0.08 ^d^	2.85 ± 0.14 ^b^	2.28 ± 0.11 ^c^	3.13 ± 0.15 ^a^
20:3 n-6	0.16 ± 0.01 ^c^	0.22 ± 0.02 ^ab^	0.18 ± 0.02 ^bc^	0.23 ± 0.02 ^a^
20:3 n-3	0.96 ± 0.04 ^d^	1.47 ± 0.06 ^b^	1.22 ± 0.05 ^c^	1.60 ± 0.07 ^a^
20:4 n-6	0.88 ± 0.02 ^a^	0.68 ± 0.03 ^c^	0.78 ± 0.02 ^b^	0.63 ± 0.03 ^c^
20:5 n-3	0.97 ± 0.01 ^a^	0.77 ± 0.01 ^c^	0.87 ± 0.01 ^b^	0.71 ± 0.02 ^d^
22:0	16.21 ± 0.17 ^a^	11.62 ± 0.23 ^c^	13.91 ± 0.20 ^b^	10.47 ± 0.25 ^d^
22:1	1.11 ± 0.01 ^a^	0.89 ± 0.03 ^c^	1.00 ± 0.02 ^b^	0.84 ± 0.02 ^d^
22:5 n-3	1.63 ± 0.08 ^a^	1.12 ± 0.04 ^c^	1.37 ± 0.06 ^b^	0.99 ± 0.03 ^d^
22:6 n-3	11.80 ± 0.29 ^a^	7.95 ± 0.39 ^c^	10.96 ± 0.39 ^b^	7.77 ± 0.43 ^c^
SFAs	53.74 ± 0.29 ^a^	49.44 ± 0.54 ^c^	51.09 ± 0.42 ^b^	49.06 ± 0.55 ^d^
MUFAs	25.82 ± 0.35 ^d^	31.58 ± 0.60 ^b^	28.70 ± 0.47 ^c^	33.02 ± 0.66 ^a^
PUFAs	5.16 ± 0.14 ^c^	5.07 ± 0.18 ^c^	6.22 ± 0.17 ^b^	7.81 ± 0.21 ^a^
HUFAs	15.27 ± 0.36 ^a^	10.51 ± 0.41 ^c^	13.99 ± 0.43 ^b^	10.11 ± 0.43 ^c^

^a, b, c, d^ Mean values in the same column with different letters are significantly different (*p* < 0.05). Data are presented as mean ± standard error values (*n* = 3). Fatty acid content is expressed as a percentage of the total fatty acid content. Abbreviations: SFAs, saturated fatty acids; MUFAs, monounsaturated fatty acids; PUFAs, polyunsaturated fatty acids (two to three unsaturated bonds); HUFAs, highly unsaturated fatty acids (four or more unsaturated bonds).

**Table 8:** Superoxide dismutase (SOD, U min^−1^), phenoloxidase (PO, U min^−1^), and glutathione peroxidase (GP_X_, mU mL^−1^) in hepatopancreatic tissue of redclaw crayfish larvae fed different experimental diets after undergoing 8 h warm air (35 °C) exposure challenge.

Treatment	SOD *	SOD ^Δ^	PO ^Δ^	GP_X_^Δ^
FM	0.08 ± 0.01	0.26 ± 0.01 ^b^	0.31 ± 0.02 ^b^	0.29 ± 0.02 ^a^
BSM-S	0.08 ± 0.03	0.21 ± 0.02 ^c^	0.26 ± 0.03 ^c^	0.24 ± 0.03 ^b^
BSM-F	0.09 ± 0.03	0.29 ± 0.02 ^a^	0.36 ± 0.03 ^a^	0.32 ± 0.03 ^a^
BSM-P	0.07 ± 0.02	0.11 ± 0.02 ^d^	0.13 ± 0.02 ^d^	0.16 ± 0.02 ^c^
Two-way ANOVA for SOD, source of variation (P > F)				
Exposure challenge		<0.0001		
Treatment		<0.0001		
Exposure challenge × treatment		<0.0001		

^a, b, c, d^ Means in the same column with different letters are significantly different (*p* ˂ 0.05). Data are expressed as mean ± standard error values (10 redclaw crayfish per tank; *n* = 3). * Before warm air (35 °C) exposure challenge. ^Δ^ After warm air (35 °C) exposure challenge. Abbreviations: BSM-S, black soldier larvae fed soybean meal; BSM-F, black soldier larvae fed fishery byproducts; BSM-P, black soldier larvae fed pitaya; FM, fish meal.

## Data Availability

The data generated in this research are reported within the article.
